# Arcuate stress state in accretionary prisms from real-scale numerical sandbox experiments

**DOI:** 10.1038/s41598-018-26534-x

**Published:** 2018-06-08

**Authors:** Mikito Furuichi, Daisuke Nishiura, Osamu Kuwano, Arthur Bauville, Takane Hori, Hide Sakaguchi

**Affiliations:** 1 Department of Mathematical Science and Advanced Technology, Japan Agency for Marin-Earth Science and Technology, 3173-25 Showa-machi Kanazawa-ku Yokohama, Japan; 2 R&D Center for Earthquake and Tsunami, Japan Agency for Marin-Earth Science and Technology, 3173-25 Showa-machi Kanazawa-ku Yokohama, Japan

## Abstract

The stress states in accretionary prisms are important for understanding the building and releasing of seismic energy. Numerous researchers have conducted sandbox experiments as a scaled physical analog model to understand the formation of accretionary prisms. However, measuring stress states in laboratory sandbox experiments is still practically infeasible. Here we performed real-scale numerical sandbox experiments using the discrete element method to understand the 3D stress state in the accretionary prism. Despite the nearly uniform initial conditions, macro-scale undulations of faults, which are similar to those observed in the trenches of an accretionary prism, appear. We reveal that these undulations are caused by the formation of stress arches. We show that the mechanism behind the arch formation is the discontinuous change in the stress orientation during the rearrangement of the stress chain. Furthermore, analyses demonstrate that the *in-situ* stress orientation from borehole data can be a signal of either the regional direction of plate convergence or the local stress orientation associated with the stress arch. The results may greatly enhance the outcome of long term monitoring in areas, such as the Nankai Trough.

## Introduction

**Figure 1:**
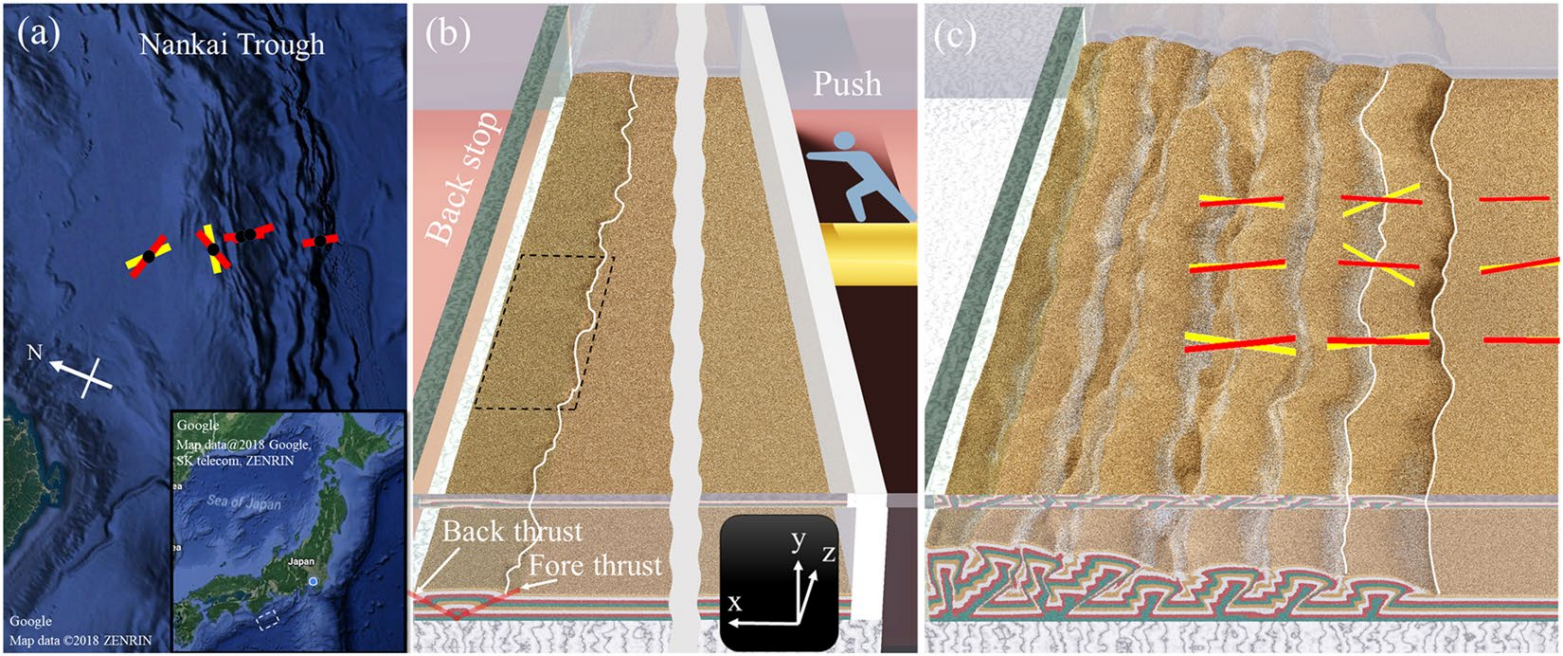
Shaded relief image of the accretionary prism at the Nankai Trough (**a**) and snapshots of the simulation results at (**b**) 1.4% and (**c**) 40% shortening. The sand particles are colored according to their initial depth (*y*-coordinate). The faults generated at the first thrust are denoted by the red lines in (**b**). The intersection of the frontal thrust with the surface is shown as a white line. The dotted line rectangle in (**b**) shows the area of the thin layer in Fig. 2. The thick colored lines are the SHmax orientations from (**a**) ocean drilling^5^ and (**c**) numerical modelling. The yellow lines represent sampling at a greater depth than the red lines. In numerical model, yellow denotes the average in 0.005 < *y* < 0.01 and the red denotes the average in 0.015 < *y* < 0.02. (**a**) was created by Google Maps^33^.

Following the great 1944 and 1946 earthquakes along the plate interface in the Nankai Trough, significant efforts have been made to monitor the stress state in an accretionary prism by the Integrated Ocean Drilling Program (IODP) and Dense Oceanfloor Network system for Earthquakes and Tsunamis (DONET) e.g^1,2^. One of the goal is to record possible changes in stress state prior to great earthquakes. The *in-situ* stress states estimated from borehole data in IODP expeditions showed significant deviations in the maximum horizontal stress (SHmax) orientation from the plate convergence as shown in Fig. 1(a) e.g.^3–5^. These deviations reflect the 3D structure of the stress state rather than the 2D structure; however the 3D stress structures as well as their effects on the evolution of the accretionary prism are not clear.

Sandbox experiments involve the horizontal shortening of a layer of sand. In these simple system, characteristic structures of accretionary prisms such as thrusts and decollements are formed. Granular motion exhibits brittle deformation similar to the deformation of rocks in the upper crust^6^. Sandbox experiments have been extensively performed to study the stress state and formation of accretionary prisms e.g.^7–9^. Despite recent methodological advances, such as the use of CT scanners to reveal the 3D inner structure e.g.^10–12^, it remains impossible to assess the stress state in physical sandbox experiments.

To overcome the limitations of laboratory experiments, we performed the real-scale numerical simulations of sandbox experiments using the Discrete Element Method (DEM) in which each grain of sand is represented by a discrete particle (Fig. 1(b) and (c))^13,14^. This method allows one to monitor the motion of each sand particle and to visualize the interparticle stress e.g.^15–18^. Stress chain analysis suggests that although the initial thickness of the sand layer varies by less than one grain diameter (i.e., micro-scale perturbation), the formation of stress arches leads to the development of macro-scale lateral undulations of faults and local deviations of the SHmax orientation from the convergence direction (Fig. 1(c)). Virtual borehole test suggests that the long-term monitoring of SHmax can be used to distinguish the local arcuate stress structure in accretionary prisms.

## Result

### Numerical simulation of sandbox experiment

The DEM assumes simple friction between rigid particles. Macro-scale deformation phenomena, such as the Mohr-Coulomb plasticity and dilation dynamically arise from this simple micro-scale behavior. In this sense, DEM is suitable for understanding basic multiscale processes by first principles. The initial configuration of the simulation comprises a granular layer with a thickness that varies by one-grain size at the most. The layer was set at 0 < *x* < 1, 0 < *y* < *h* and 0 < *z* < 1 where the unit length is m. This system size is consistent with the typical size of laboratory experiments e.g.^11^. Gravity is applied in the negative *y*-direction. The initial layer is surrounded by walls and the right-hand side wall and bottom wall were pushed in the *x*-direction toward the backstop wall at *x* = 1 at a constant velocity of 0.1 m/s. The walls perpendicular to the *x*- and *y*-axis (bottom, fore, and backstop walls) have low friction coefficients and the side walls in the *z*-direction satisfy the free-slip condition. Details of the initial configuration and DEM are given in the Methods section. Next, we present simulation results with particle radius 〈*r*_*p*_〉 = 227.5 µm, where 〈·〉 denotes the average value, and 241 million particles fill a layer of initial thickness *h* = 1.88 cm. The model parameters are summarized in Table 1.

**Table 1:** DEM parameters.

Particle radius *r*_*p*_	227.5 ± 22.5 µm, (113.75 ± 11.25 µm, 455.0 ± 45.0 µm in Fig. 5)
Particle density *ρ*	2,600 kg/m^2^
Yang modulus *E*	1 × 10^7^ Pa
Poisson ratio *m*	0.2
Coefficient of friction *μ*	0.6 (0.3 ~ 3.0 in Fig. 4 and Supplementary Fig. S2)
Coefficient of friction *μ*: walls	0.25: walls against x- and y-direction, 0: walls against z-direction
Coefficient of restriction *e*	0.2
Coefficient of rolling friction *μ*_*r*_	0.05
Number of particles	240,960,960: 241 M, (30 M ~ 1.9B in Figs 4 and 5)

The range of parameters in the validation tests in Fig. 4 and Supplementary Fig. S2 with different thickness and *μ*, and the convergence test with different particle sizes in Fig. 5 are in parentheses.

When pushed against the backstop, the sand layer deforms into pop-up structures with new frontal thrusts forming sequentially, a behavior of typical taper wedges formed in the sandbox experiments (Supplementary Movie 1). The situation after the formation of the first pop-up structure is shown in Fig. 1(b). The frontal thrust of the first pop-up undulates. At the end of the experiment, the undulation of the frontal thrust in Fig. 1(c) is similar to the geometries seen in accretionary prisms in Fig. 1(a). Both the amplitude and characteristic length of the thrust undulation are several orders of magnitude greater than the grain size. To understand the manner in which the initial micro-scale perturbations of the layer thickness produce macro-scale scale structures, we performed a stress chain analysis.

### Evolution of the stress state inside the granular layer

**Figure 2:**
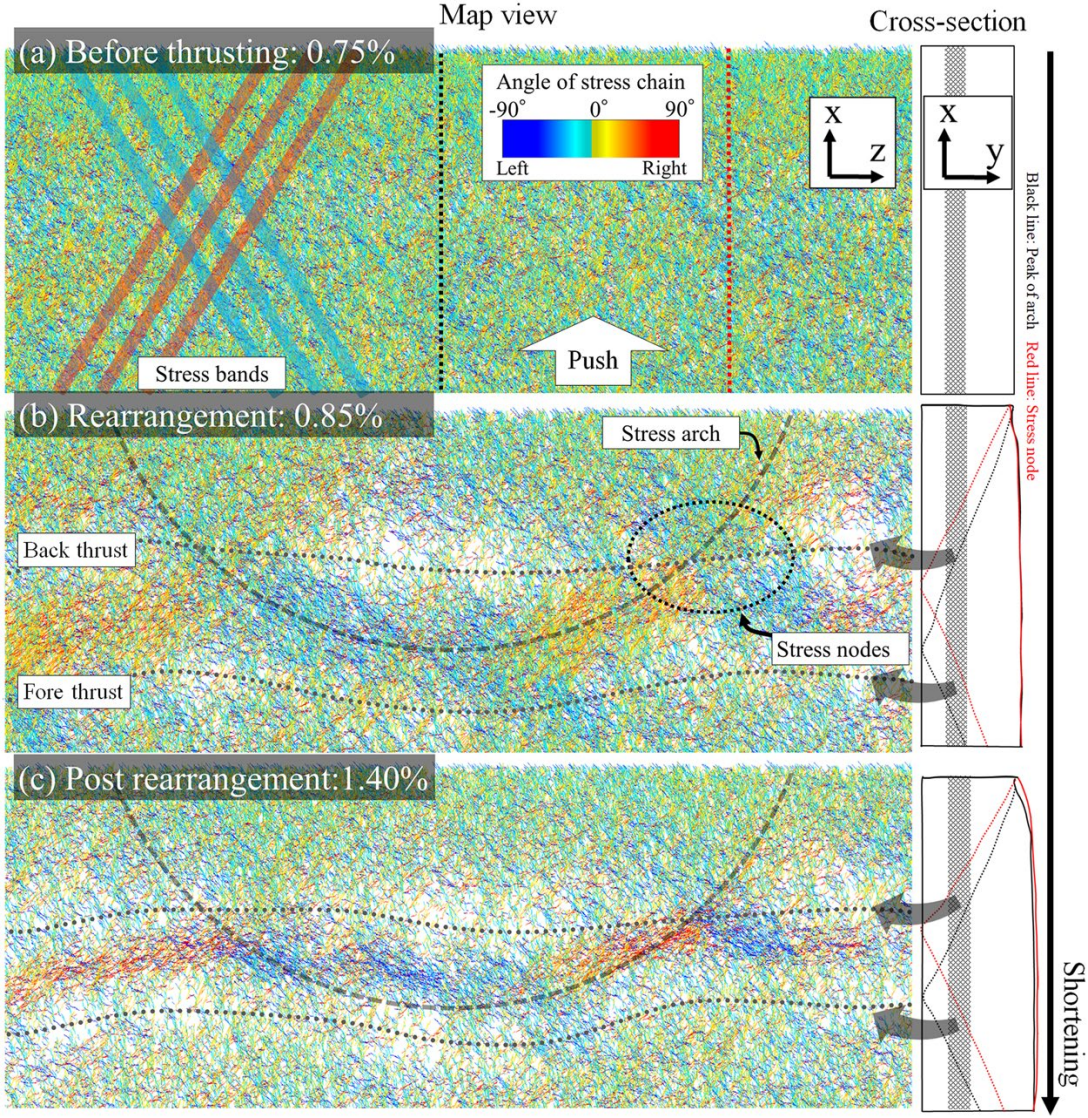
Evolution of the stress chains during the first thrust formation. The degree of the shortening is (**a**) 0.75% before thrusting, (**b**) 0.85% at the rearrangement, and (**c**) 1.4% at the post rearrangement. Map views of the most compressive principal stress vector in the stress chain in the thin layer (0.94 < *x* < 1, 0.005 < *y* < 0.01, and 0.35 < *z* < 0.52) are shown. The colors denote the angle of the vector from the *x*-axis in the horizontal plane. Cross-sectional views of the thrust at the peaks of the arch (black dotted line) and the stress nodes (red dotted line) are also shown (black and red dotted line in the map view (**a**)) at the righthand side of the map view. The thin layers and thrusts in the map are denoted by the shaded area and the thick arrows in the cross-sectional view, respectively.

We performed a stress chain analysis of the first thrust event. A stress chain is the co-linear arrangement of particles carrying a load that is larger than the average load (see Method section). To quantify the stress chain, we used the method proposed by Peter *et al*.^19^. The map views of the stress chains and cross-section views of the geometry at three different shortenings are shown in Fig. 2. In the map views, the most compressive principal stress directions for particles belonging to the stress chain within the thin layer are represented as colored lines for the angle in the horizontal plane. Cross-sectional views of the thrust system are also illustrated.

Soon after the beginning of the simulation, the stress chains intensify in the shortening direction (*x*-direction) (Supplementary Movie 2). A multitude of stress bands are oriented ~30° from the *x*-direction in the *xz*-plane (Fig. 2(a)). This angle can be predicted by the macroscopic friction angle of the sand. With shortening, a pop-up structure bounded by a fore and back thrust is formed. In Fig. 2(b), the faults correspond to regions where stress chains have largely disappeared in comparison to those in Fig. 2(a). In the thrust, the particles become disconnected and existing stress chains break. Simultaneously, the reconnection of particle contacts induces the rearrangement of the stress chains in and around the pop-up. At the beginning of the rearrangement stage, thick bands are generated. Then, positively and negatively tilted thick bands cross the back thrust and generate the nodes. In the meantime, in the pop-up structure, pairs of positively and negatively tilted bands connect.

The stress chain structure in the rearrangement stage has the shape of an arch in Fig. 2(b). The horizontal undulations emerge at the same time as the stress arch. The undulation of the frontal thrust is consistent with the shape of the arch. As shown in the cross-sectional views of Fig. 2, the positions of the thrusts differ in the arch region, because the compressional strength is maximum at the peak of the arch. Detailed observations of the cross-sections are discussed in Supplementary Sec. S1.

After the rearrangement stage, the active thrust kept uplifting the pop-up structure. In this post rearrangement stage, the arch structure gradually changes but keep its signature, as shown in Fig. 2 (c). The undulation of the thrust survives and characterizes the pop-up structure in the lateral direction.

**Figure 3:**
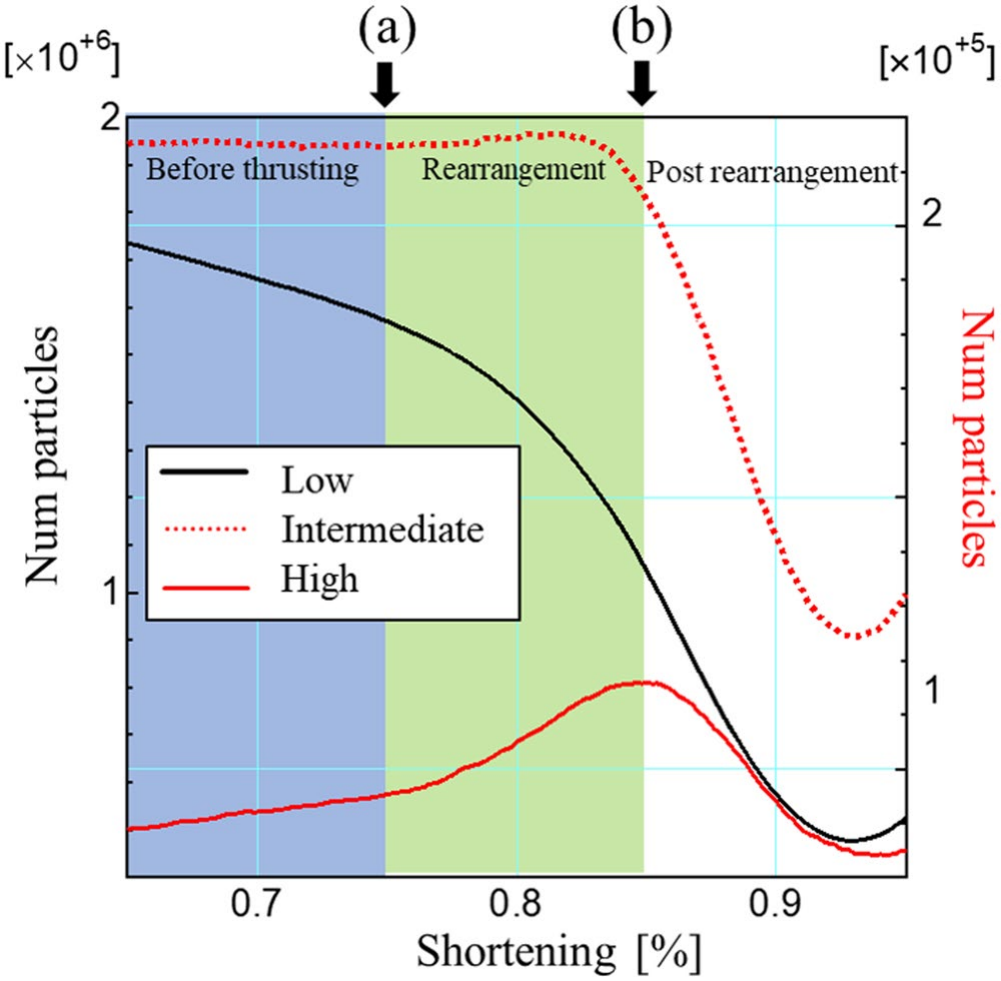
Population of particles within the stress chains for different ranges of angle *θ*_*p*_ of the stress vector against the *x*-axis in the *xz*-plane in the region of 0.94 < *x* < 1 and 0.05 < *z* < 0.95. The particles are classified as low-, intermediate- and high-angle with *θ*_*p*_ < 40°, 40° ≤ *θ*_*p*_ < 60°, and *θ*_*p*_ ≥ 60°, respectively. The low-angle and other categories are plotted using the left and right axis, respectively. The stages are denoted by the thick arrows at the top.

The stress orientation characterizes the initiation of the thrust in Fig. 2 (also Supplementary Sec. S3); thus, we analyze the evolution of angle *θ*_*p*_ between the most compressive principal stress direction within a stress chain and the *x*-direction. The particles are grouped into *θ*_*p*_ < 40°, 40° ≤ *θ*_*p*_ < 60°, and *θ*_*p*_ ≥ 60° and are referred to as low-, intermediate- and high-angle groups, respectively. Figure 3 shows the number of particles in each category as a function of shortening. Before thrusting, the number of particles in the low-angle category decreases, while the population of high-angle particles slightly increases. Thrusting triggers the rearrangement stage, and decrease in low-angle particles and increase in high-angle particles are accelerated. The low-angle stress chains are disrupted by the change in the particle contacts in the thrust zone and high-angle chains emerge in the popup to construct the arch peak. The intermediate category has almost constant number of particles up to around the end of the rearrangement stage. Thus, the number of stress chains shift from low- to high-angles but not through the intermediate-angle group. Such a discontinuous behavior is out of the general continuum behaviors and characterizes the nonlinear process behind the generation of undulations in the frontal thrust. At the post rearrangement stage, the populations of the stress chains decrease in all categories owning to active thrusting, while the arch structure of the stress chains survives.

### Factors controlling the stress arch geometry

To understand the factors controlling the arch geometry, we applied the bridge-curve model^20^. We used the parabolic arch model by assuming that the first arches were generated to bear the uniform compressive loads in the *x*-direction (see Method section). To identify the parabolic curve of the arch, the following two boundary conditions are needed: the distance between the peak position of the arch and the backstop, and the angle of the chains at the backstop wall. From the results of the DEM simulation, we estimated them by the layer thickness *h* (initial geometry) and angle of the thrust *θ* (macroscopic material property) (Supplementary Sec. S1). The width of the parabolic arch model is calculated as follows:

1 $${z}_{d}=\frac{h{\cos }^{2}\theta }{\sin \,\theta (1-\,\sin \,\theta )}$$

**Figure 4:**
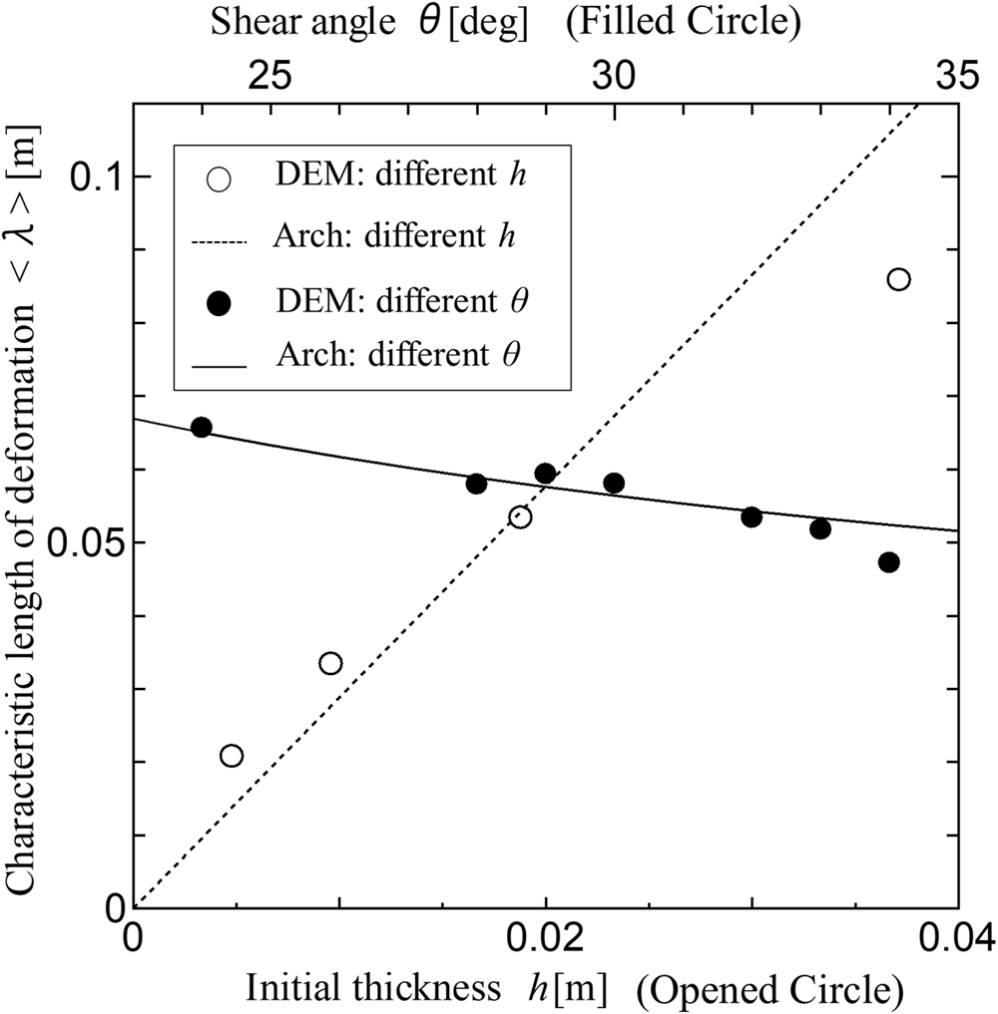
Comparison of the results of the parabolic arch model and DEM simulations. The length of the arch structure is plotted against the contacting friction angle *θ* in the top axis and the initial thickness of layer *h* in the bottom axis. Characteristic length of deformation in DEM calculations was estimated by the DFT analysis (see Method section). The length of the parabolic arch model is given by Eq. (1).

In Fig. 4, we compare the lateral deformation length by the DEM simulations and the width of the arch by the theoretical models for variable thickness *h* and angle *θ*. The angle *θ* of the simulation depends on interparticle friction coefficient *μ* (Supplementary Sec. S2). We apply the discrete Fourier transform (DFT) to the length scale of the horizontal deformation (see Method section). Despite the lack of full agreement, the DEM simulations are reasonably consistent with the model result.

**Figure 5:**
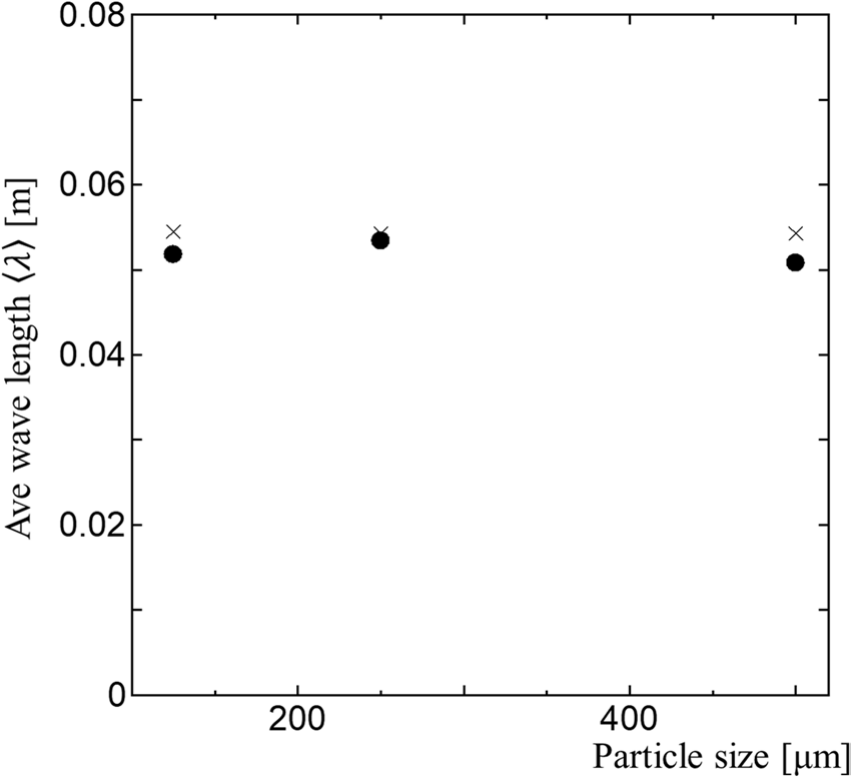
Comparison between the DEM simulation (filled circles) and the parabolic model (crosses) for different particle sizes (*r*_*p*_ = 113.8 μm, *r*_*p*_ = 227.5 μm, and *r*_*p*_ = 455.0 μm, computed by 4,096 CPUs, 432 CPUs, and 48 CPUs of the K-computer, respectively). The DFT wavelength 〈λ〉 cuts off the deformation less than about eight particle sizes for different 〈*r*_*p*_〉 (see the Methods section).

To check the effect of grain sizes to the arch geometry, we varied the particle sizes in the experiments, and run the simulations up to 1.9 billion particles with 〈*r*_*p*_〉 = 113.8. For all particle sizes, the same angles of thrust (*θ* = 32°) are observed. In a previous study, the peak shear strength was similarly insensitive to the particle size^21^. Figure 5 shows the effect of grain sizes. The consistency between the DFT wavelength and the model suggests that the arch length does not depend on the element size.

These numerical results justify the use of the layer thickness and the angle of thrust as the first-order controlling factors with the parabolic arch model.

### Stress chains in the late thrust event

**Figure 6:**
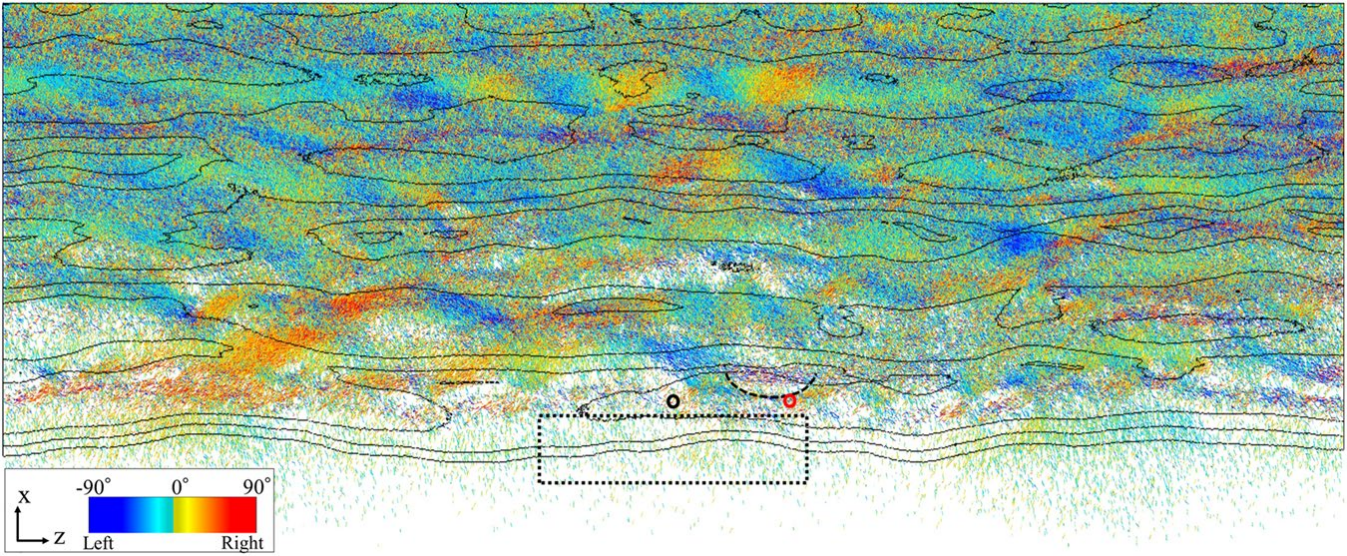
Stress chain distribution in the layer 0.01 < *y* < 0.015 at 40% shortening. The most compressive principal stress vectors in the stress chains are denoted by the colored lines using the same colors as in Fig. 2. The black lines mark the contour lines of the height of the granular material. The black and red circles denote the position of Hole-A and Hole-B, respectively, in Fig. 7. The dotted rectangular area is used to evaluate the activity of the thrust. The dashed line illustrates the stress arch involving the Hole-B.

The stress state at the later thrust formation is more complex than the first event (Supplementary Movie 3). We plot the stress chains and surface topography at the eighth thrust event in Fig. 6. Some of the early formed arch structures are obscured by the deformations caused by the late thrusts. The undulation of the frontal thrust is not always generated by the stress arch, but it is generated by the salient of the earlier formed thrust. Nevertheless, the lateral heterogeneities, including the stress arches, are clear.

**Figure 7:**
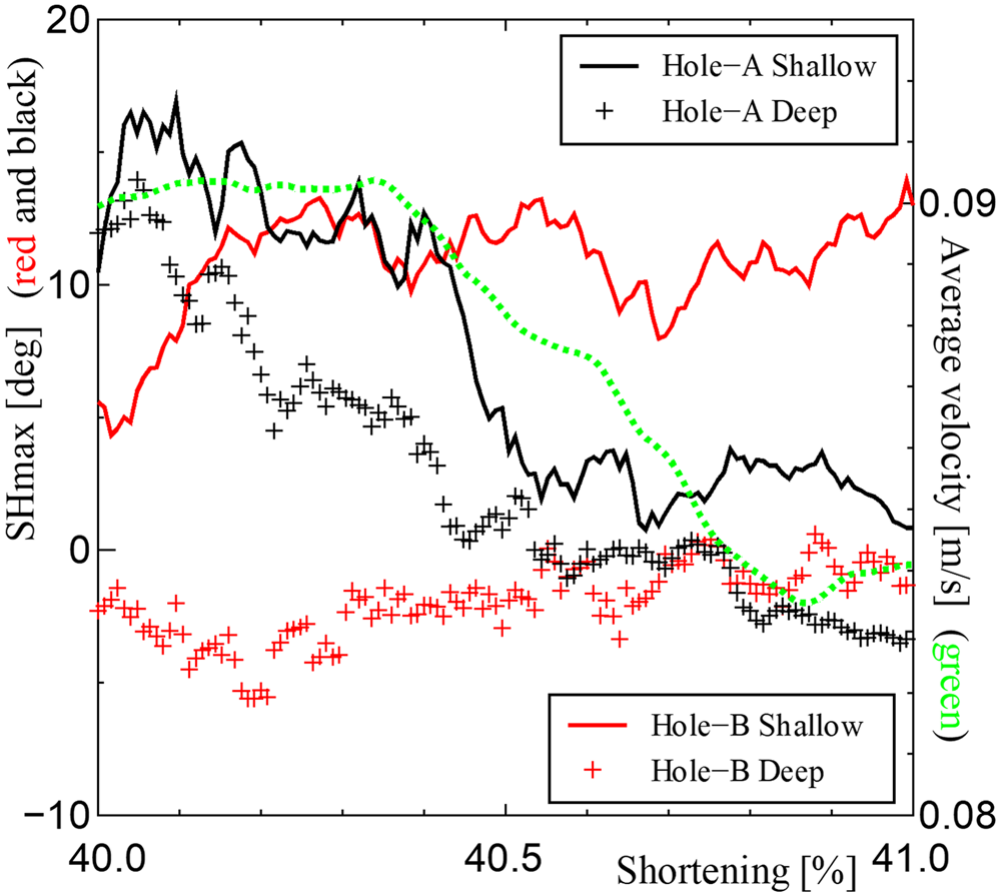
SHmax orientation from the numerical boreholes and thrust activity. The SHmax orientation is given by the averaged principal stress vectors in the cylinder centered at Hole-A (black) and Hole-B (red) with a radius of 5 mm. The positions of Hole-A and Hole-B are illustrated by the circles in Fig. 6. The line and cross show the angles of SHmax from the x-axis at the shallow part (0.01 < *y* < 0.015) and deep part (0.005 < *y* < 0.01). The thrust activity is defined by the average velocity at 0.64 < *x* < 0.69 and 0.4 < *z* < 0.6 shown in Fig. 6.

We examined the manner in which the horizontal stress arches after multiple thrust events were recorded in the boreholes. Figure 7 shows the evolution of the orientation of SHmax averaged over all the particles, including those that are not in the stress chains, in the layers of two numerical boreholes (Hole-A, black and Hole-B, red) of Fig. 6. We define the orientation of SHmax as the direction of the most compressive principal stress vector on the *xz*-plane because SHmax is significantly large when the stress field is compressive enough to generate a reverse fault^22^. Hole-A and Hole-B are located outside and inside the stress arch, respectively. The averaged particle velocity of the frontal thrust area near the boreholes (dotted rectangular area of Fig. 6) was also monitored, as the regional activity of the thrust. In Hole-A, the orientations are similar in both deep and shallow areas. The orientations decrease in accord with the regionally averaged velocity. Thus, the stress orientation in Hole-A probably reflects the overall stress state of the pop-up that is controlled by the thrust activity. However, the SHmax orientation inside the arch (Hole-B) comprise positive and negative angles depending on the depth. Similar rotation is also seen in the orientation of the stress chains beneath the arch structure (Supplementary Sec. S4). The evolution of the orientation in Hole-B does not correlate with the thrust activity, but it is rather constant and maintains the local arch structure. These results suggest that the factors controlling the stress orientation vary significantly in two closely spaced boreholes.

## Discussion

One of the key findings is that arcuate faults emerge from micro-scale perturbations owning to the formation of the stress arch. With an analogy to the deformations between the upper crust and the granular material, this observation leads to a new feasible mechanism for generating arcuate geological-scale (km-scale or higher) structures from micro-scale perturbations without geological-scale perturbations, such as topography, layer property, and erosion^23–25^. To validate this hypothesis in nature, the first place is to confirm the relationship between the surface undulations, material properties and the depth profile of subduction zone, which are measurable by the geophysical observations and experiments. For example, Eq. (1) predicts 6–60 km undulations in 1–10 km-thick sediments under an angle of thrust *θ* = 30°. Further discussion at the specific sites should involve the initial geological-scale irregularities and surface processes because not all curves in the thrust belt were formed in this manner^26^.

The stress concentration to generate the stress arch seems not to be specific to the granular simulation. By resolving the micro-scale inhomogeneity, the mesh-based numerical simulation can also capture the stress concentration^27^. We expect that a continuum model with a proper constitutive law can generate an arcuate stress pattern from small random noises in a self-organizing manner. This idea will be explored in our future work.

Another important observation is that the complex lateral stress structure is a natural outcome of the multiple thrust events (Fig. 6). We argue that such stress states improve the interpretation of the stress data from the boreholes. 2D cross-sections of the sandbox experiments have been used to interpret borehole data e.g.^7,16^; however, thus far, 3D views have not been used well. For example, the numerical borehole experiment shows the deviation and rotation of the SHmax from plate convergence (Fig. 1(c)). Such behavior is consistent with *in-situ* stress data by the ocean drilling e.g.^3–5^ (Fig. 1(a)). Lateral stress state inhomogeneities have been discussed as the effect of tectonic anomalies, such as sea mountains^3,28^. However, the simulation results suggest that the stress inhomogeneities are also attributable to the inherent nonlinearities in crustal deformation (Fig. 6). The long-term monitoring of the SHmax orientations in boreholes may support this hypothesis if the evolution of stress state is controlled by the local arcuate stress structure, as shown in Fig. 7.

Our analysis also suggests that the thick tilted stress band moves laterally with the propagation of the thrust (Supplementary Movie 3). Therefore, the release of stress parallel to the trench axis by structures hidden inside the pop-up is probably linked to the lateral succession of seismic events, such as those observed in the Nankai Trough.

## Conclusions

Direct numerical simulations of the sandbox experiment suggest that the macro-scale lateral stress structures, such as arcuate structural patterns are due to micro-scale perturbations. Stress chain analysis reveals that the particles collectively form such structures owning to the discontinuous changes in the stress chains at the beginning of the fault motion. Similar multiscale behavior is expected to generate the complex 3D stress states inside accretionary prisms without geological-scale perturbations. We also demonstrated that 3D sandbox simulations can help to understand the lateral stress orientations and their evolutions from borehole data.

## Methods

### DEM model

In this section, we describe the physical model and setup of the sandbox experiments in detail. DEM is a first-principles calculation method for granular dynamics calculations based on the motion of individual particles. Newton’s equations of motion for individual particles are as follows:

2 $$M\frac{\partial {\boldsymbol{u}}}{\partial t}={{\boldsymbol{F}}}_{{\rm{c}}}^{{\rm{n}}}+{{\boldsymbol{F}}}_{{\rm{c}}}^{{\rm{t}}}+M{\boldsymbol{g}}$$

3 $$I\frac{\partial {\boldsymbol{\omega }}}{\partial t}={r}_{p}\,{\boldsymbol{n}}\times {{\boldsymbol{F}}}_{{\rm{c}}}^{{\rm{t}}}-{\mu }_{r}r\{1-{({\boldsymbol{n}}\cdot {\boldsymbol{\omega }})}^{2}/{{\boldsymbol{\omega }}}^{2}\}|{{\boldsymbol{F}}}_{{\rm{c}}}^{{\rm{t}}}|{\boldsymbol{\omega }}/|{\boldsymbol{\omega }}|$$

where ***u***, ***ω***, *M*, *r*_*p*_, and *I* are the particle translational velocity, angular velocity, mass, radius, and moment of inertia, respectively. ***n*** is the unit normal vector to the contact point between particles from the particle center. *μ*_*r*_ is the rolling friction coefficient. ${F}_{{\rm{c}}}^{{\rm{n}}}$ and ${F}_{{\rm{c}}}^{{\rm{t}}}$ are the normal force and tangential force induced by the contact between two particles or a particle and a wall. These contact forces are represented using the Voigt model, which comprises a non-linear spring and dashpot and stems from the Hertz–Mindlin model^29,30^.

4 $${{\boldsymbol{F}}}_{{\rm{c}}}^{{\rm{n}}}={K}_{ij}^{{\rm{n}}}{{\boldsymbol{\delta }}}_{ij}^{{\rm{n}}}+{\eta }_{ij}^{{\rm{n}}}{{\boldsymbol{v}}}_{ij}^{{\rm{n}}}$$

5 $${{\boldsymbol{F}}}_{{\rm{c}}}^{{\rm{t}}}={K}_{ij}^{{\rm{t}}}{{\boldsymbol{\delta }}}_{ij}^{{\rm{t}}}+{\eta }_{ij}^{t}{{\boldsymbol{v}}}_{ij}^{{\rm{t}}}$$

where ***δ***_*ij*_ is the relative displacement vector and ***v***_*ij*_ is the relative velocity vector between particles *i* and *j*. In the normal direction, the spring constant of each contact point between particles *i* and *j* is determined according to the Hertzian contact theory

6 $${K}_{ij}^{{\rm{n}}}=\frac{2E}{3(1-{m}^{2})}\sqrt{\frac{{({r}_{p})}_{i}{({r}_{p})}_{j}}{{({r}_{p})}_{i}+{({r}_{p})}_{j}}|{{\boldsymbol{\delta }}}_{ij}^{{\rm{n}}}|},$$

which depends on the normal compressed length $|{{\boldsymbol{\delta }}}_{ij}^{{\rm{n}}}|$ of the spring. *m* is Poisson’s ratio and *E* is the Young’s modulus of the particles used in the DEM. In the tangential direction, the spring constant is represented as follows:

7 $${K}_{ij}^{{\rm{t}}}=\frac{4G}{(2-m)}\sqrt{\frac{{({r}_{p})}_{i}{({r}_{p})}_{j}}{{({r}_{p})}_{i}+{({r}_{p})}_{j}}|{{\boldsymbol{\delta }}}_{ij}^{{\rm{n}}}|},$$

where *G* is the shear modulus which is related to the Young’s modulus and Poisson’s ratio as follows:

8 $$G=\frac{E}{2(1+m)}$$

and consequently

9 $${K}_{ij}^{{\rm{t}}}=\frac{3(1-m)}{(2-m)}{K}_{ij}^{{\rm{n}}}.$$

The dumping coefficient *η* is represented as follows:

10 $${\eta }_{ij}=\frac{1}{2.2}\,\mathrm{ln}(\frac{2}{e}-\,1)\sqrt{\frac{{M}_{i}{M}_{j}}{{M}_{i}+{M}_{j}}{K}_{ij}}.$$

wherer *e* is the restitution coefficient. Eq. (10) is associated with the non-linear spring model^31^. If the tangential force by the Voigt model satisfies the following relation:

11 $$|{{\boldsymbol{F}}}_{{\rm{c}}}^{{\rm{t}}}| > \mu |{{\boldsymbol{F}}}_{{\rm{c}}}^{{\rm{n}}}|,$$

particles slide between each other and the tangential force is given by

12 $${{\boldsymbol{F}}}_{{\rm{c}}}^{{\rm{t}}}=\mu |{{\boldsymbol{F}}}_{{\rm{c}}}^{{\rm{n}}}|{\boldsymbol{t}}$$

where the kinetic friction force is based on the Coulomb-type friction law instead of the spring and dashpot. *μ* is the friction coefficient and ***t*** is the unit tangential vector. Note that the restitution (viscous damping) force owning to the dashpot and the kinetic friction force act as dissipative forces in terms of the inter-particle contact force. In addition, we consider the rolling friction effect in the second term of Eq. (3)^32^.

One of the difficuties of DEM simulations is that the number of particles, system size, and grain size are limited by the computational cost. Thus, it is essential to use parallel computing; nevertheless, the efficient parallel implementation of DEM remains a challenge. We developed new DEM codes designed for HPC systems to overcome the degradation of parallel performance due to the load imbalance by utilizing an iterative dynamic load balancer^13^ and overlapping communication techniques with space-filling curves. The details of the parallel implementation method are discussed in^14^.

In the sand box setup, the initial granular layer is created using the free-fall calculations from the uniformly distributed particles in the grid cell, with very small random perturbation velocities. The radius of the particles is randomly selected from 0.9 < *r*_*p*_/〈*r*_*p*_〉 < 1.1. The friction coefficient is set to zero for dense packing. From this initial condition, we start the product runs with the parameters in Table 1. The friction coefficient of the boundary walls in the *x*- and *y*-directions are set small *μ* = 0.25 and represent the décollement behavior.

### Stress chain analysis

Stress chain analysis is a powerful tool to reveal the non-continuum features of granular dynamics. Stress chains are seen in laboratory experiments with photo-elastic granular materials and are generally thought to linkage the most compressive principal stress vectors of each particle that have magnitudes larger than the average value. Although stress chains are seen in the large principal stress vectors, such images are too complex to analyze because most of the large stress vectors are not part of the stress chains. Thus, identifying stress chains is important for analyzing the complex 3D dynamics of granular materials. We quantitatively identify the stress chains via the method proposed by^19^. Thus, we first calculate the most compressive principal stress vector (***σ***_3_) from the particle stress tensor as follows:

13 $${\sigma }_{IJ}=\frac{1}{V}\sum _{c=1}^{{n}_{c}}\,{f}_{I}^{c}{r}_{J}^{c}$$

where 1 < c < *n*_*c*_ denotes all particle pairs in contact, *V* is the volume of a particle, *f*_*I*_ is the *I*-th component of the force between two contacting particles, and *r*_*J*_ is the *J*-th component of the radius vector from the center of the particle to the point of contact with the paired particle. Tension is positive value in Eq. (13); thus, the most compressive vector is the minor principal stress ***σ***_3_. To form the stress chains, *i*-th particles should have $|{{\boldsymbol{\sigma }}}_{3}^{i}|$ greater than the average and should contact *j*-th particles. In addition, the stress vectors of the *i*-th and *j*-th particles (i.e. ${{\boldsymbol{\sigma }}}_{3}^{i}$ and ${{\boldsymbol{\sigma }}}_{3}^{j}$) should be aligned in the relative position vector ${{\delta }^{n}}_{ij}$ within a 45° angle. Finally, we count the length of the strongly connected particles and identify the stress chains that are longer than a given number of particles, which is four in this case.

### Parabolic arch model

The arch structures in the *xz*-plane are formed owning to the tightly connected particles that bear the compression in the *x*-direction. Here, we consider the arch structure centered at *z* = 0 with the force balance in the *xz*-plane and neglect the gravity in the *y*-direction for simplicity. The arch structure is assumed to be a string applying uniform pressure gradient *w* in the *x*-direction.

When the tension of the arch at the peak point is *T*_0_, the compression of the string of the arch can be written by $(T\,\sin \,{\phi }_{s},T\,\cos \,{\phi }_{s})=(wz,{T}_{0})$ where *z* is the distance from the center of arch, and *φ*_*s*_ is the tangential angle of the curve of the string. Then, we can derive the equation for the arch shape as follows:

14 $$\frac{dx}{dz}=\,\tan \,{\phi }_{s}=\frac{w}{{T}_{0}}z$$

**Figure 8:**
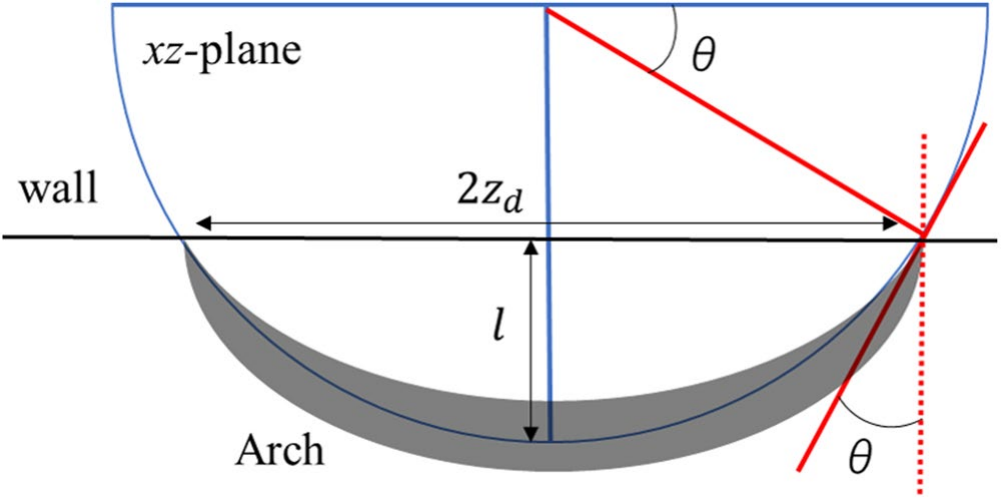
Schematic of the parabolic arch model.

The solution of Eq. (14) is the parabolic function as follows:

15 $$x=\alpha {z}^{2}+\beta $$

where *α* = 2*T*_0_/*w* and *β* are constants to be defined by two boundary conditions that are the angle of the chains at the back boundary (back stop wall) and peak height of the arch. Figure 8 shows the model arch structure at the first thrust near the backstop wall. The height of arch is $l=h/\tan \,\theta$ and is the distance between the backstop wall and center of the pop-up structure. Since the thick stress bands tend to align with the angle of thrust *θ*, the orientation of the stress bands stacked to the backstop wall is assumed to be the angle *θ*.

From these two boundary conditions, we obtain the arch length [i.e. Eq. (1)]. From this expression, we can deduce the length scale of the stress arch in nature.

### DFT analysis

**Figure 9:**
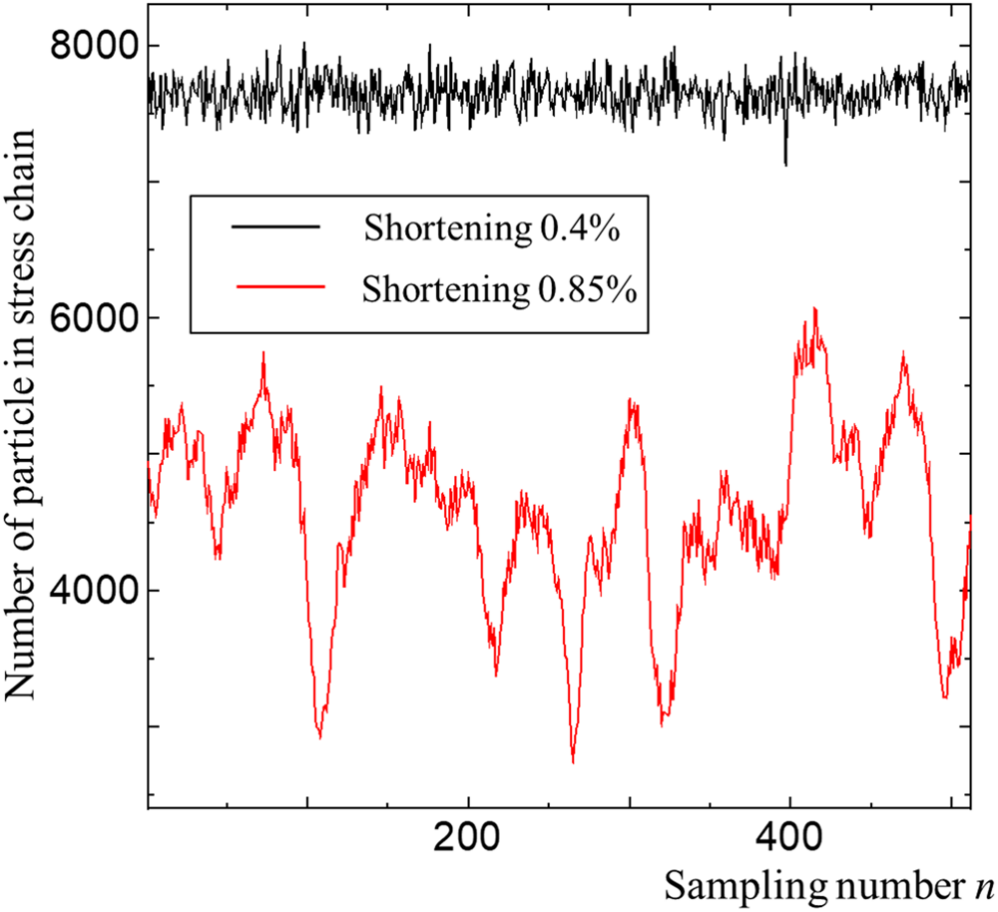
Distribution of stress chains sampled along the *z*-axis.

The stress chain distributions along the z-axis characterize the horizontal deformation of granular media; thus, we use Discrete Fourier Transform (DFT) to quantify the length scale of the horizontal deformation. We focus on the deformations in the first thrust close to the backstop wall and avoid the side boundary walls. We analyze the distribution of particles in the stress chain for 0.95 < *x* < 1 and 0.05 < *z* < 0.95. The discrete signals *P*(*j*) with sampling number n = 512 of stress chain distributions projected onto the *z*-axis at 0.4% shortening before onset of the thrust and 0.85% shortening after the onset of the thrust are shown in Fig. 9. The chain distribution is white noise at the early stages of the simulation; however, several large peaks appear at the node of stress band after the thrust formation. To quantify the length scale of the peaks characterizing the lateral curved structure, we use the DFT as follows:

16 $$A(k)=\sum _{j=0}^{n-1}P(j){e}^{2\pi ijk/n},$$

Then we calculate the averaged wavelength, as follows:

17 $$\langle \lambda \rangle =\frac{{\sum }_{k=1}^{N-1}\,\frac{0.9}{k}|A(k)|\,}{{\sum }_{k=1}^{N-1}|A(k)|\,}.$$

where the summation run is over *N* − 1 from *k* = 1 and focuses on the deviation from the average, i.e., the value at *k* = 0 is omitted. The sampling number *N* in Eq. (17) is a model parameter used to focus on the macro-scale granular deformation while dumping the grain-size scale deformation < ~(*L*/*N*) where *L* = 1. We use *N* = 256 for the wavelength analysis for 〈*r*_*p*_〉 = 227.5 µm. The average wave length 〈*λ*〉 with *N* = 256 does not consider micro scale deformation less than about eight particles because 8 × 〈*r*_*p*_〉 ≈ (1/256). We confirme that the wavelengthes from Eq. (17) in Fig. 4 are almost the same when we used different sampling window with 0.1 < *z* < 0.9.

However, the DFT analysis suffers from leakage errors because the observed curve is not purely periodic and limitation of the window size. The poor frequency resolution with a phase discrepancy between the start and end of the sampling data reduces the peak amplitude at the target frequency. Thus, the large wavelength at the initially thick layer in Fig. 4 is underestimated. Increasing the sampling window improves leakage problem.

## Electronic supplementary material

Supplementary information

Movie 1

Movie 2

Movie 3
