# Supplementary material for: Arcuate stress state in accretionary prisms from real-scale numerical sandbox experiments
Source: Sci Rep. 2018 Jun 8;8:8685. doi: 10.1038/s41598-018-26534-x (PMC5993738; doi:10.1038/s41598-018-26534-x)
Supplement: Supplementary file 1 — Supplementary information [file 41598_2018_26534_MOESM1_ESM.docx]

**Supplementary information of**

**“Arcuate stress state in accretionary prisms from real-scale numerical sandbox experiments”**

**Mikito Furuichi^1^*, Daisuke Nishiura^1^, Osamu Kuwano^1^, Arthur Bauville^1^, Takane Hori^2^ and Hide Sakaguchi^1^**

**^1^** Department of Mathematical Science and Advanced Technology, Japan Agency for Marin-Earth Science and Technology 3173-25 Showa-machi, Kanazawa-ku Yokohama, Japan

**^2^** R&D Center for Earthquake and Tsunami, Japan Agency for Marin-Earth Science and Technology 3173-25 Showa-machi, Kanazawa-ku Yokohama, Japan

Correspondence to Mikito Furuichi [m-furuic@jamstec.go.jp].

**S1. Cross-section of the pop-up structure**

The arch structure increases the compressive strength and produces rheological anisotropies on the macroscale. To explore the role of the arch in the horizontal deformation, cross-sections of the particle distributions in the node and peak regions (Fig. 2(c)) are shown in Figs. S1. The colors denote the deviation of the height from the initial particle position. The green and yellow regions represent the shear bands. The same angle of the front thrust in Fig. S1 suggest that the angle $\theta$ depends on the macroscale material property based on such as the Mohr–Coulomb failure criterion.

At the node region (Fig. S1(b)), the edge of the back thrust is close to the backstop wall. However, at the peak region (Fig. S1(a)), the back thrust forms further away from the backstop. The thrust positions reflect the rheological strength of the arch structure. Consequently, the positions of the frontal thrust between the node and peak differ. This observation justifies correlations between the stress arch structures and the undulation of the frontal thrust.

In the simulations, as in laboratory experiments [e.g. 34], numerous weak shear bands emerge before the localization of the deformation in the thrust. The light blue color region in front of the frontal thrust in Figs. S1 contains weak shear bands. The weak shear bands at the node position (Fig. S1(b)) are wider than those in the peak region (Fig. S1(a)) because stress localization is affected by the arch structure. This numerous short-lived weak shear bands are proposed to control the fluid migration in accretionary prisms [34, 35].


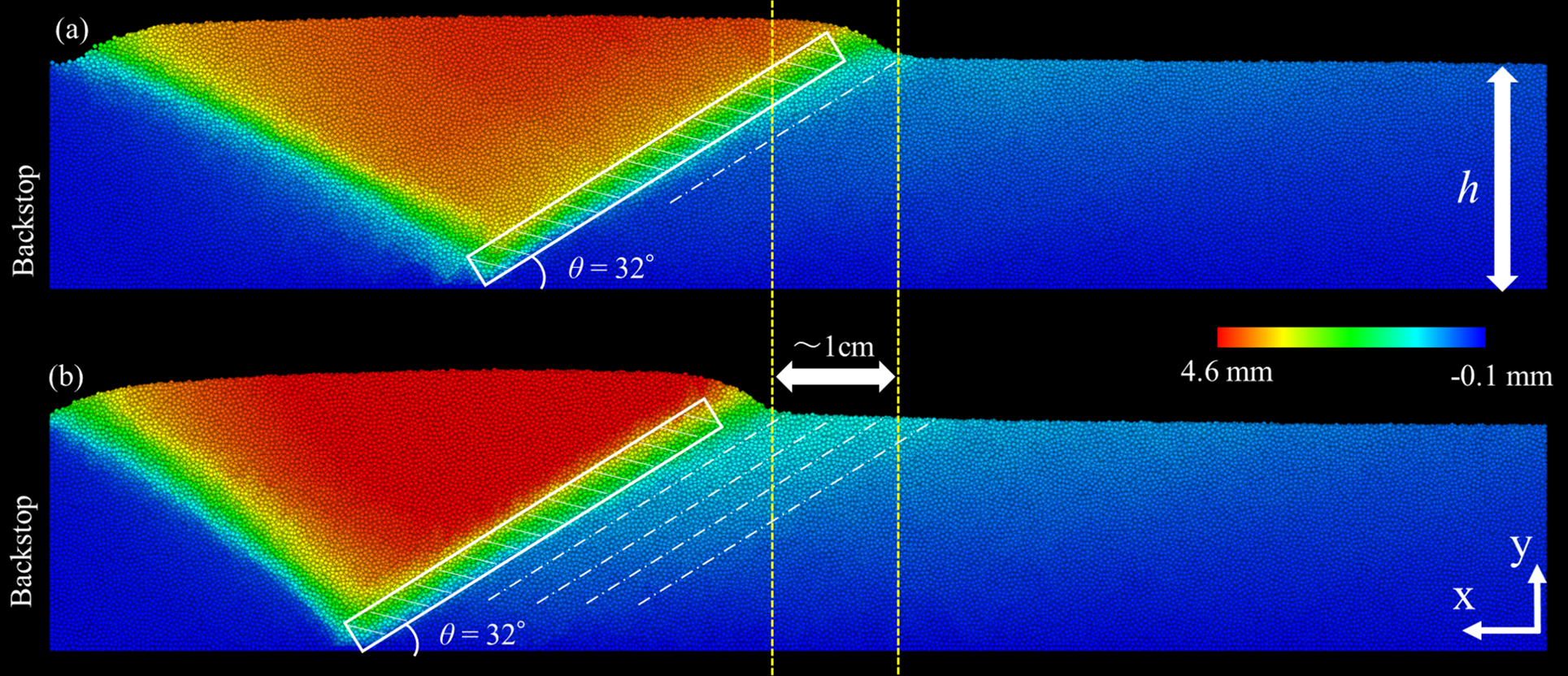


Figure S1: Cross-sectional view of the particles in the thrust at the (a) peak and (b) node of the stress arch in Fig. 2. The colors denote the commutative displacement of the particle height from the initial position. The shape of the shear band is delineated by the white box to measure the angle of thrust $\theta$. The weak shear bands are shown by the white dot-dashed lines.

**S2. Contact friction and the thrust angle.**

In Fig. 4, we controlled the angle of thrust $\theta$ with the contact friction $\mu$ because the increase in contact friction is known to increase the bulk strength of granular materials [36] . Figure S2 shows the angle $\theta$ for different contact friction coefficients. The DEM model successfully reproduces the monotonical decrease in the angle $\theta$ with an increase in the friction coefficient. The gradient of angle $\theta$ seems to change at around $\mu=0.6$. The detail comprehensive mechanism for this nonlinearity requires further investigations.


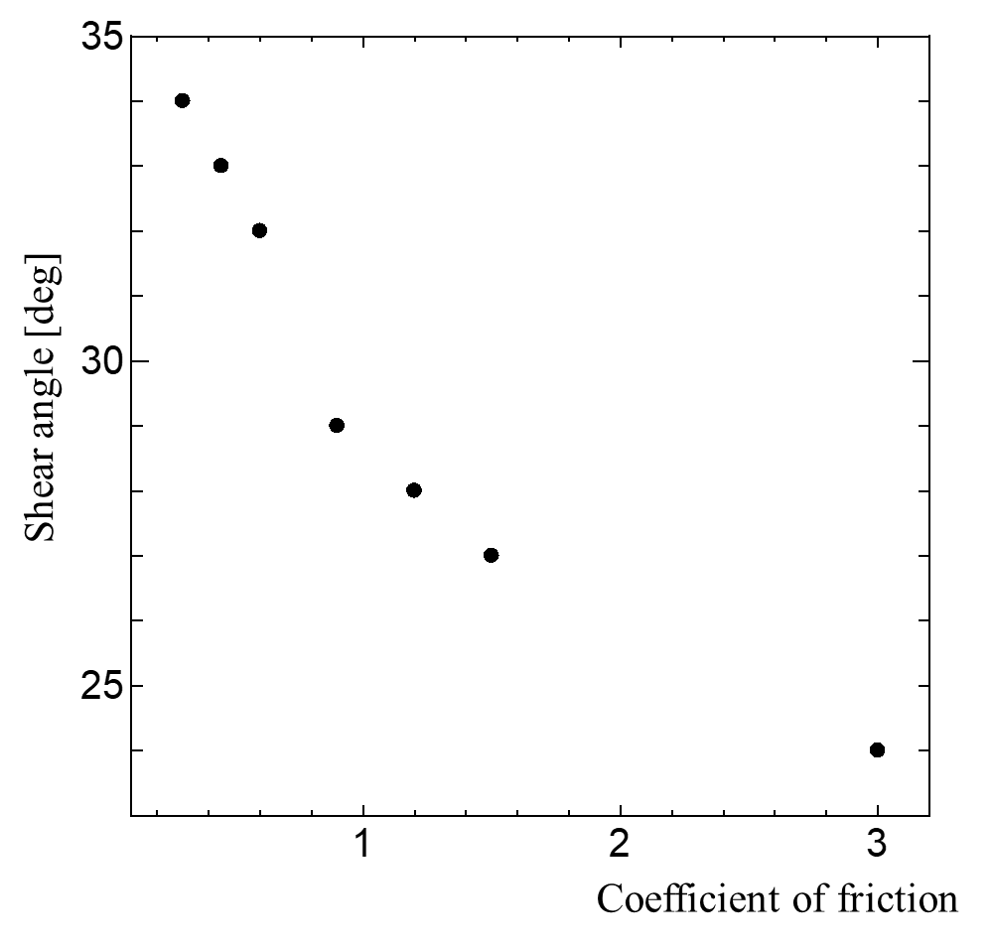


Figure S2: Angle of thrust against contact friction angle.

**S3. Length scale of lateral deformation and chain angle**

The change in the connection of particles in the fault zone under shear induces the rearrangement of the stress chain. The orientation of the principal stress vector during this rearrangement stage is important for generating the arch as shown in Fig. 2. The low angle chains in the *x*-axis shift to the high angle chains in the pop-up to construct the peak of the arch. Figure S3 shows the increment of the averaged angle of the stress chains $\Delta\theta_{k}=\left\langle\theta_{p} \right\rangle_{k}-\left\langle\theta_{p} \right\rangle_{k-1}$ at the *k*-th output timestep and the averaged wavelength $\left\langle\lambda\right\rangle$ of DFT analysis for the stress chain distribution along the *z*-axis (Sec. M3). The wavelength $\left\langle\lambda\right\rangle$ is used as an indicator of the length scale of the deformation. A drastic increase in angle was started at the onset of thrust and was lasted until the first peak of $\Delta\theta$ at around *t* = 0.085 s, when the structure of arch essentially forms. The similarly scaled curves in Fig. S3 suggest that the horizontal deformation that generates the undulation of thrusts depends on the principal stress orientations.

Figure S3: Comparison between the average wave length of the DFT and the angle increment of the stress chain.


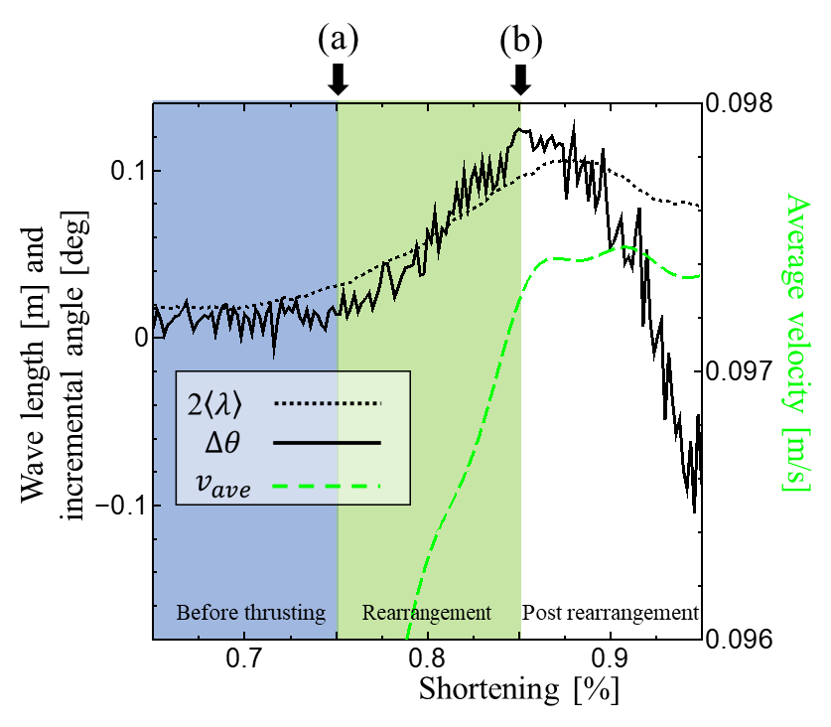


**S4. Stress chains at depth**

To understand the stress arch structure in the *y*-direction, Figs. S4(a) – S4(d) show the stress chains of the thin layers at 0.9% shortening for $0.94 \leq x<1.0$, $0.35 \leq z<0.52$ and (a)$0.01 \leq y<0.015$, (b)$0.005 \leq y<0.01$, (c) $0.002 \leq y<0.005$ and (d)$0 \leq y<0.002$. The thrusts are the bands allined in the *z*-direction, where the stress chains are less dense than the surroundings. The pop-up between the fore and back thrusts becomes thin with an increase in depth and disappears at the bottom layer (Fig. S4 (d)). In Figs. S4(a) and S4(b), similar arch structures with stress nodes at the same position in the *x*-axis are observed. Since the upper layer suffers less load than the deeper layer, the population of stress chains in Fig. S4(a) is less dense than Fig. S4(b). The chain near the bottom has more complex structures than that in Figs. S4(a) and S4(b). The shape of the arch in Figs. S4(c) and S4(d) is not clear from the chains in the deep layers. The width of the pop-up is too thin to construct the peak of the arch bridging the two stress nodes (red dotted lines) because stress chains with high curvature are not formed owning to the limitations of the frictional connection. In addition, the stress arch seems to control the lateral geometry of the thrust because the undulation of the thrust at the bottom layer of Fig. S4(d) is consistent with the arch structure. The change in the color with the red circle region from Figs. S4(b) to S4(c) suggests the rotation of the chain orientation with depth in the pop-up.


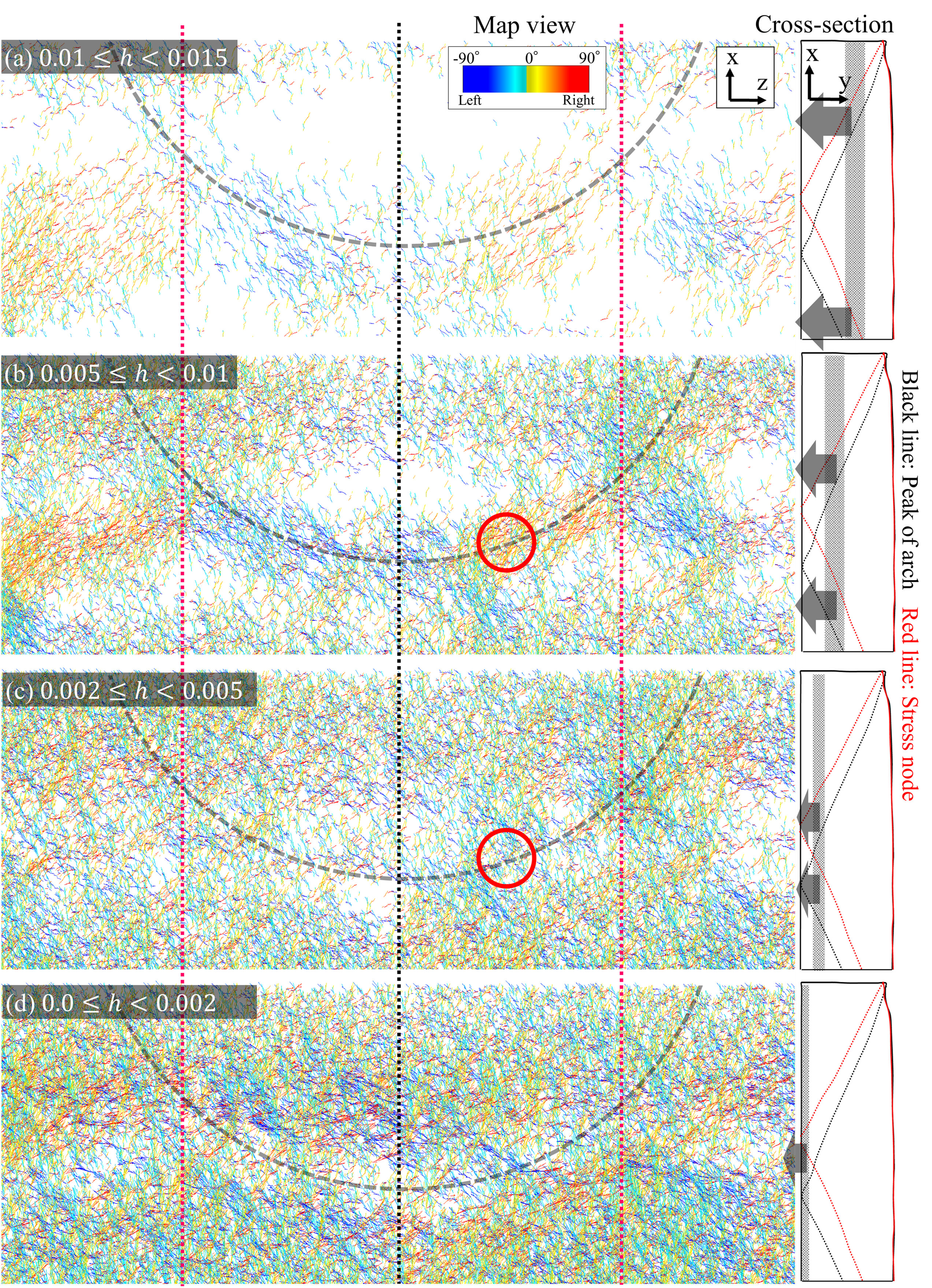


Figure S4: Map views of the most compressive principal stress vector in the stress chain of the thin layer for $0.94 \leq x<1.0$ and $0.35 \leq z<0.52$ at different depth (a)0.01 ≤ *y* < 0.015, (b) 0.005 ≤ *y* < 0.01, (c) 0.002 ≤ *y* < 0.005 and (d)0.0 ≤ *y* <0.002 at 0.9 % shortening. The position of the peak of the arch and the stress node are shown by the black and red lines, respectively. The cross-sectional views of the thrust at the peak of the arch and stress nodes are also shown. The layers used in the map are shown by the shaded area in cross-section. The lines and color map are the same as in Fig. 2. The rotation of the stress chain orientation is denoted in the red circle.

**References**

[34] Dotare, T., Yamada, Y., Adam, Hori, T., & Sakaguchi, H. Initiation of a thrust fault revealed by analog experiments. *Tectonophysics* **684**, 148-156 (2016).

[35] Yamada, Y., Baba, K., Miyakawa, A., & Matsouka, T. Granular experiments of thrust wedges: Insights relevant to methane hydrate exploration at the Nankai accretionary prism. *Mar. Pet. Geol.* **51**, 34-48 (2014).

[36] Thornton, C. Numerical simulations of deviatoric shear deformation of granular media. *Geotechnique* **50**, 43-53 (2000).

**Supplementary Video File information**

Movie 1: Video of the sandbox simulation result shown in Fig. 1(b) and Fig. 1(c).

Movie 2: Video of the stress chain evolution during the first thrust formation shown in Fig. 2. The lines and color map are the same as in Fig. 2.

Movie 3: Video of the stress chains in the late thrust events shown in Fig. 6. The lines and color map are the same as in Fig. 6.
